# Strategies to Inspire Students’ Engagement in Pharmacology Courses

**DOI:** 10.3390/pharmacy9020070

**Published:** 2021-03-26

**Authors:** Hussein N. Rubaiy

**Affiliations:** Department of Health and Caring Sciences, Faculty of Health and Occupational Studies, University of Gävle, 801 76 Gävle, Sweden; hussein.rubaiy@hig.se; Tel.: +46-(26)-648500

**Keywords:** pharmacology, student engagement, higher education, health professional

## Abstract

Pharmacology is a distinct discipline and offers core knowledge to broaden student programs in the provision of health care (medicine, nursing, pharmacy, and others) as well as research-oriented programs (biosciences and biomedical). Therefore, knowledge and information on topics such as prescribing medication, drug interactions, dosage adjustments, and the correct drug dose calculation for medicine are essential for preventing and reducing medication errors, which is a key element in patient safety. Recently, many institutions have been trying to enhance their quality of teaching, as students demand support and success in their studies. Pharmacology is a highly challenging interdisciplinary topic, and requires a huge effort from both educators and students to achieve the best outcomes. Therefore, creating an effective environment to support students’ learning in pharmacology courses is essential to improving students’ engagement, success and learning outcomes. In recent decades, the landscape of education has changed, and distance learning has skyrocketed. This manuscript intends to discuss and highlight the importance of student engagement in higher education in pharmacology courses. Additionally, this paper spotlights and presents a review of recent studies focusing on student engagement in pharmacology courses and possible methods for enhancing and inspiring student engagement in pharmacology courses.

## 1. Introduction

Recently, many universities, faculties, institutions and educators in all disciplines have been trying to enhance their quality of teaching as students demand support and success in their studies. Therefore, creating effective environments to support students’ learning in higher education is essential to improving student success and retention. Pharmacology is a distinct discipline, and offers core knowledge such as drug–receptor interactions, mechanisms of drug action, drug distribution, metabolism, and therapeutics for broad health care programs such as medicine, pharmacy, and nursing. Moreover, pharmacology is a demanding interdisciplinary topic in biosciences and health professions that requires a huge effort from both sides—educators and students—to achieve the best outcomes within a course. Importantly, pharmacology is essential for students in health care disciplines (pharmacists, physicians, nurses, and others) who are looking to establish themselves in improving patient care, which has become a priority for all health care providers, and also for those who have a particular interest in improving drug therapy and making it safe for the patients’ benefit. This review discusses and highlights research papers and reviews that discuss student engagement in pharmacology courses in health care programs.

## 2. Student Engagement

For almost three decades, student engagement has drawn attention in the literature, illustrating the importance of student engagement as a crucial component of teaching in higher education [1]. Student engagement can be summarized as active student participation inside or outside of the classroom that results in improved learning experiences and outcomes. Plenty of studies in higher education and sufficient evidence in recent years have highlighted the importance of student engagement, regardless of discipline, suggesting that students’ active engagement in classes will benefit their learning [2,3,4]. Therefore, to improve and build passionate learning habits among students, it is necessary to generate a classroom environment that encourages good student practices. Some of the points related to fostering student engagement include: (a) providing students with an awareness of different forms of research-mindedness and scholarly approaches to learning, (b) promoting the active use of various forms of self-assessment in students, and (c) encouraging students to see themselves as active producers of knowledge, and not as passive consumers [5,6,7].

A simple question that needs be answered is: why should students be engaged? In other terms, what are the effects of engagement in teaching in higher education? Rush and Balamoutsou conclude that [8] “Engaged students … share the values and approaches to learning of their lecturers; spend time and energy on educationally meaningful tasks; learn with others inside and outside the classroom; actively explore ideas confidently with others; and learn to value perspectives other than their own. When students are part of a learning community … they are: positive about their identity as a member of a group; focused on learning; ask questions in class; feel comfortable contributing to class discussions; spend time on campus; have made a few friends; and are motivated in some extra curricular activity”.

**Figure 1:**
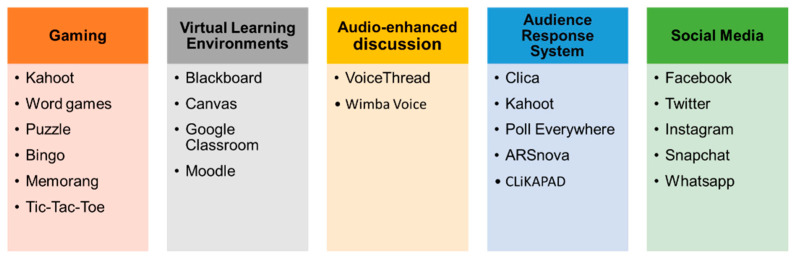
Five pillars of digital tools (FPDT) to enhance student engagement, motivation, and activity.

There are numerous types of digital tools available to instructors to be integrated with academic activities, an approach that aims to shift learning to a more student-centered, rather than teacher-centered, model in order to promote student engagement [9]. These various tools can be grouped into gaming, virtual learning environments, audio-enhanced discussion, audio response systems, and social media (Figure 1), and each of these will be discussed in further detail below. Figure 1 highlights the five pillars of digital tools (FPDT) that are used to enhance engagement, motivation, and activity among students within higher education. Educators integrate digital tools such as virtual learning environments, gaming, and social media into their academic activities, as today’s students are more reliant on technology for learning than previous generations [9,10,11,12,13,14,15,16,17,18] (Figure 1).

The vast majority of today’s student body grew up with digital technology, and own a smartphone. According to the biannual Mobile Survey Report (2018), of the 4135 participating college students, almost all students, 99.8%, owned a mobile device and 82% used a mobile app for learning at least once each week [19]. The report also showed that students within health care programs such as medicine and nursing use smartphones for learning more than other student groups (Figure 1) [19]. Furthermore, almost half of the participating students, 40%, wished that their instructors would use mobile apps and devices within the courses. On the basis of this evidence, instructors should try to incorporate the use of digital tools more in order to promote students’ learning, while, at the same time, increasing their motivation, activity and overall engagement (Figure 1) [9,18].

Nevertheless, student engagement does not happen by itself in any discipline, as students, staff, local context, institutions, and educational ideology are the critical success factors for engagement [1]. To improve student engagement, The University of Guelph in Canada [20] has taken on a three-part strategy aiming to empower students to take control of their learning, increase their engagement in research and learning and, most importantly, to encourage students to develop skills which enable them to selectively process and transform information into knowledge and wisdom. The three parts, which take place both outside and inside the classroom, include (1) fostering community engagement and citizenship by encouraging first-year students to volunteer in the local community, (2) offering a variety of courses and extracurricular activities to foster service learning and volunteering, and lastly (3) offering first-year students the opportunity to register for an interdisciplinary course taught in a small-group format, including enquiry-based learning (EBL) and problem-based learning (PBL). Those who participated in these two types of group performed better than those who did not [20]. It is suggested that students who engage in the three strategy parts in their first year of university studies seem to develop skills which improve their ability to learn, and were encouraged to become more involved with the community, which altered the pattern of access to learning sources. With this said, the students achieved better retention, better satisfaction and better academic performance, in both qualitative and quantitative terms [20].

## 3. Student Engagement in Pharmacology Courses

Pharmacology education is required in various programs within higher education including medical and nursing schools, schools of pharmacy, dentistry, physiotherapy, and veterinary medicine, as well as research-oriented biomedical programs. This review focuses on major pharmacology courses for medical, pharmacy, and nursing students. Certainly, pharmacy, medical, and nursing teaching faculties are aware of the challenges which both educators and learners face in pharmacology courses [21,22,23,24].

With a vast majority of students using digital devices continuously during the day and increasingly using these devices for learning, there is a growing interest in gamification, and its usage in teaching pharmacology to engage the students [13] (Table 1). Game-based learning has been shown to be fun and interactive, as well as to facilitate learning. This type of learning also provides a learning environment that creates challenges, curiosity, immediate feedback with rewards and low-stake failure [18]. There are games such as word games, puzzles, tic-tac-toe and others to help improve pharmacology knowledge among students. A study regarding this topic showed that these types of games improved students’ acquisition of pharmacology knowledge (63.5%), improved student participation (61.9%) and improved self-learning (74.6%) [13] (Table 1). An additional useful tool when teaching pharmacology is the implementation of audience response systems (ARS). A study revealed that using ARS compared to traditional face-to-face teaching increased students’ motivation to learn, and exams’ correct rates rose from 73.3% to 90.0% [12] (Table 1).

Furthermore, a popular way to revise and learn pharmacological concepts as well as engaging students is to use the game-based learning platform Kahoot [10,15] (Table 1). Kahoot is an interactive game in which the instructor can create a number of multiple-choice questions that are relevant to the learning objectives. The students log into the Kahoot game through their mobile phones or computers using a specific code for the game. The questions are shown on the classroom projector, and students answer either individually or in small groups by choosing one of four options. The benefits of Kahoot are that students receive immediate feedback and it creates a friendly competition among students. According to a study by Sumanasekera et al., students believed that using Kahoot to learn pharmacological concepts was the most valuable active learning strategy, compared to fill-in-the-blank activities and videos [15] (Table 1).

Another point is that research- and inquiry-based teaching and learning can mean a number of things, which will not be discussed in this manuscript; however, they are essentially defined as using research to frame your teaching [34,35,36]. In brief, the nature of undergraduate research and inquiry can include the following: (A) research-tutored, where students are engaged in research discussions; (B) research-based, where students undertake research and inquiry; (C) research-led, which is learning about current research in the discipline; (D) research-oriented, which is developing research and inquiry skills and techniques [34,36,37]. Undoubtedly, the enhancement of student engagement inside and outside of the classroom or in an online classroom environment will enable and facilitate students to learn difficult pharmacological concepts. However, student engagement in higher education in any discipline, particularly in large class sizes within pharmacology courses, remains a dilemma.

## 4. Medical Students

In recent years, universities have been focusing on the integration of basic science and clinical applications to improve their undergraduate medical curriculum [38]. Medical students need to receive a thorough education in understanding the pharmacology that is needed for effective pharmacotherapy and prescribing of medication. Studies have shown that newly qualified doctors lack strong knowledge of drugs, causing thousands of medication errors [24,39,40,41]. Therefore, those responsible for medical schools have recently implemented various strategies with the aim of improving medical students’ knowledge of pharmacology.

Zeheib et al. [30] (Table 1) stated that using team-based learning (TBL) to teach pharmacology improved the group performance compared with individual performance. However, their study also revealed that, when the questions were very difficult, the TBL was less successful [30] (Table 1). Combining newly learned information and the use of primary research articles enhanced student engagement in the teaching of second-year medical students. Moreover, students learned considerably more, particularly when the information was largely novel [34].

In a separate study, blended learning, which combines the online digital media with traditional classroom methods, was used to assess the student engagement in a clinical post-graduate students’ pharmacology course [32,42] (Table 1). Traditional classroom learning is a method where a teacher controls and regulates the flow of information and knowledge (by teaching face-to-face), and the students are anticipated to continue increasing their knowledge of a subject outside of the class [42,43]. The participating undergraduate students were keen on the use of blended learning in their pharmacology course, although they wished that the blended learning was supported by tutorials and very structured with high quality. Furthermore, the students acknowledged that this sort of teaching was more beneficial and helpful compared to solely online learning [32] (Table 1).

Currently, several computer-based pharmacology systems are offered on the internet or are commercially available. Kerecsen and Pazdernik gave an in-depth review of 30 years of experience in the computer-aided instruction of pharmacology [31] (Table 1). They reported that the constant necessity to update, with respect to both contents and changing computer technologies in medical education, are the major challenges [31] (Table 1). However, utilizing computers can be helpful in many different ways and depends significantly on the nature of the curriculum for the course.

Digital tools have also been introduced to various higher education programs, including the use of social media. On average, an internet user spends 145 min every day on social media sites, which creates a good opportunity for instructors to reach students easily [44]. One study by Quesnelle and Montemayor showed the benefits of using Facebook posts within medical programs to promote student engagement [33] (Table 1). The posts which generated the greatest student engagement were question-type posts. Quesnelle and Montemayor concluded from the study that the use of Facebook can create virtual learning communities that can combine formal and informal learning [33].

## 5. Nursing Students

All nursing programs comprise pharmacology content, either integrated throughout the program or as a stand-alone course. Generally, the pharmacological concepts are difficult for nursing students to grasp; therefore, educators have designed and implemented strategies to encourage and facilitate students’ understanding and comprehension of nursing pharmacology. As evidence, pharmacology training for nursing students has been claimed to be insufficient [45,46].

In recent years, a number of novel strategies to teach pharmacological content and medication administration have been developed: (1) scavenger hunt—students retrieve and record information related to foundational pharmacological concepts, such as nomenclature, pharmacoeconomics, pharmacokinetics, pharmacodynamics, and ethical considerations, using over-the-counter (OTC) medications [47]; (2) the village—to determine the pathophysiology of diseases and the medications required to treat the patients, students utilized hypothetical families and case studies [22]; (3) the medication mansion—in order for students to learn and memorize medications, a visual approach was applied, using sticky notes placed in different rooms around the house [48]; (4) a psychopharmacology conceptual handout [49].

Blended learning within nursing has been shown to favor greater achievement of effective learning when implemented with a Learning Management System (LMS) and Virtual Learning Environments (VLE), such as Blackboard, Canvas and Moodle, together with hypermedia resources consisting of graphics, audio, video, plain text and/or hyperlinks. These types of platforms allow students to access information at any time, which is not possible when interacting face-to-face. They also allow students to easily personalize their learning, leading to increased student motivation [25] (Table 1).

An increasing number of studies show that there is a significant gap between knowledge and implementation of skills in pharmacology content and drug-dosage calculations among nursing students and practicing nurses [50]. One strategy taken to strengthen nursing course content is the recruitment of pharmacists to work with the nursing students. Work sessions involving the simulation of real-life problem-solving have increased nursing students’ confidence to collaborate more with, and benefit from, pharmacists’ expertise in the clinical area [50]. Another positive strategy has been to assess students throughout the term by giving biweekly quizzes covering lecture material solely for that period, which are not cumulative over the course. The average result of the best five quiz results out of seven would account for 25% of the total grade. A final exam covering all course material would account for 35%, and the remaining 40% of the grade would involve clinical application quizzes. This type of course assessment has increased student success and satisfaction with course content. It is vital for students to receive a strong foundational knowledge in pharmacology, calculations, and medication administration techniques to enable them to be successful in their future workplace [50].

Thomas and Schuessler reported numerous factors that contributed to poor outcomes in pharmacology teaching for a baccalaureate nursing program when pharmacology was taught in a traditional classroom manner and not through student-focused techniques [26] (Table 1). These factors were as follows: (1) students feared the consequences of not being successful on the pharmacology specialty exam, (2) the amount of information given was overwhelming and difficult to retain, and (3) students were disengaged in the learning process [26]. Remarkably, student engagement, learning outcomes, and the exam scores were improved through the application of various instructional strategies such as games, case studies, and humor [26]. In more detail, using games and case studies improved the students’ engagement compared with the lecture-only approach in nursing programs, which generally struggles to keep the students engaged in the pharmacology course [26]. In a separate study, Vana et al. reported that students’ understanding or retention of pharmacological knowledge did not improve using multiple-choice PowerPoint slides and an audience response system compared with those who solely used multiple-choice PowerPoint slides [51].

Carpenter et al. stated that nursing students generally memorize information within a pharmacology course, rather than developing their comprehension of how to administer safe medication in a clinical setting [52]. At the end of their pharmacological studies, nursing students are required to have an understanding of therapeutic uses, drug dosages, side effects, drug–drug interactions, precautions and contraindications. The nursing faculty at Lake College in the US state of Louisiana implemented a number of steps to facilitate an improvement in students’ retention. Pre-lecture sessions held on Fridays aimed to help students prepare for the following week’s class lectures, instructing students on how to use the textbook effectively. A couple of days after the lecture, a post-lecture recitation is held, to recap and clarify the covered pharmacology material. This post-lecture session offers an opportunity to expose any problem areas, reiterate ideas or redirect, if needed. Further introduced measures are pre- and post-examination reviews. The Friday prior to examination, students play revision games, which are an effective teaching tool, work in small competitive groups and are able to take charge of their learning needs while having fun. Immediately after the examination, a session is held in which the answers of the multiple-choice questions are read, with the sole purpose of decreasing the students’ anxiety about their grade. As separate measures, pharmacology instructors record videos and post a web-based quiz on the content that is going to be covered in the next class session. In addition, the instructor has the option of creating a blog covering the content. These online strategies triggered students to stimulate the development of their clinical imagination. These steps have encouraged students to be more prepared for class and, consequently, class retention significantly increased [36].

In 2015, Bata-Jones and Avery compared two different approaches to teaching nursing students: the web-based and face-to-face pharmacology course in nursing [53]. Eighteen students were picked to participate in the web-based course, and 52 chose to participate in the face-to-face course. The same educator taught both courses. Interestingly, the results showed no significant differences between the registered students’ mean examination scores in the two course types. However, the students in the online course were positive about the course and highlighted the need for clarity of instructions, and the amount of information provided on the course website [53].

Correct drug-dose calculation is an essential skill to prevent and reduce medication errors, which is a key element for patient safety. The literature suggests that drug-dose calculation within a pharmacology course is challenging for nursing students [46,54]. To understand the weak knowledge, which could be a result of deficiencies in basic education, or a lack of ongoing maintenance training during working years, Simonsen et al. compared the medication knowledge, certainty and risk of error between graduating bachelor students in nursing and experienced registered nurses [46]. This work, which was a multiple-choice test on pharmacology, drug management and drug-dose calculations, compared bachelor students in closing term and registered nurses with at least one year’s work experience. Participants comprised 243 graduating students and 203 registered nurses, with an average working experience of 12.4 years. From their study, they concluded that the medication knowledge among experienced nurses was superior to bachelor students in nursing; however, it was still not sufficient [46]. This work and others highlight the important of drug-dose calculation among nursing students and experienced registered nurses.

Undoubtedly, the comprehensive nursing program is deeply laden with the learning content; therefore, nursing students are challenged by a content-laden curriculum. Equally, the nurse educators, who strive to accomplish the program’s heavy curriculum, are facing the challenge of supporting the nursing students’ success and outcome by delivering a student-centered learning approach. Kaylor, who studied undergraduate pharmacology in a nursing course, reported the use of cognitive load theory (CLT) as an instructional design framework [27] (Table 1). The CLT, which was established by John Sweller (late 1980s), claimed that instructional design could possibly be used to decrease the cognitive load for students.

Currently, there are various approaches to including gaming in nursing education. Studies have shown that gaming enhances the teaching and remediation of content, as well as being fun, improving motivation and promoting student engagement. Examples of games include “Are you smarter than a nursing instructor?” or pharmacology bingo [11] (Table 1). Another interesting tool among the FPDT is the use of audio-enhanced discussion boards. These boards, such as VoiceThread, enable the inclusion of various media, allowing student participation through text, audio, and web commenting. A report by Reyes et al. showed the benefits of this type of board when nursing students practiced communicating with imaginary patients about not-so-easy diagnoses, such as a positive pregnancy test for a teen [14].

## 6. Pharmacy Students

With the increasing number of technological teaching tools and various teaching methods, educators must be aware that there is not a “one-size fits all” approach. Pharmacy educators need to consider which methods and tools are suited to their groups. This is essential, as pharmacy students are presented with a large amount of information over a short period of time (2–3 years of didactic), making it more difficult for students to succeed (31). Recently, Oyler and his colleagues offered an in-depth review of pharmacist educators regarding student engagement in pharmacy school [28] (Table 1). The review presents the reader with practice philosophies to enhance the engaging learning environment for pharmacy students. It is worth mentioning that Oyler et al. also discussed the “chunk and chew” method to improve student engagement and the learning outcome. In this approach, the educators split a subject into subtopics (chunks) and permit the students to process (chew) the material in groups between sections [28] (Table 1).

In recent years, PowerPoint slides have increasingly grown in popularity compared to the more traditional chalk-and-talk lecture design. Notably, this approach to teaching works very well; however, in a pharmacy curriculum, the importance of the basics is emphasized, suggesting that the PowerPoint slides method is not the only method that can achieve the best learning outcome [29] (Table 1). Therefore, in pharmacy schools, to improve the student engagement, a combination of PowerPoint slides with chalk-and-talk was designed in a pharmacology lecture on diuretics [29] (Table 1). This case study reported that, in addition to traditional PowerPoint slides, utilizing the active development of concept diagrams encouraged student engagement and enhanced the understanding of content in pharmacology lectures at pharmacy schools [29] (Table 1).

Studies have shown that an estimated 30% of students at a distance site either talk or sleep during class sessions, and only half are willing to ask questions, as many have microphone anxiety and are worried about interrupting a peer at another site. One method to improve students’ involvement during class is to implement an audience-response system (ARS). An investigation into the usage of ARS systems across three pharmacy campuses showed that a large majority, 85.3% of students, felt that the ARS facilitated class participation, and 75.7% reported that their class focus increased during synchronous video teleconferencing sessions. ARS systems can also be implemented during on-site class sessions (34).

Another tool to help increase student interaction during sessions on one campus or multiple campus sites is a wiki tool. This tool allows not just one student at a time, but an unlimited number of students, to post answers, comments and questions on a virtual wall. Users collaborate to create, edit, or delete content. The educator can create a column for each case or discussion question, allowing students to post their comments. This allows educators to receive responses from more students than the one or two students who typically respond during a traditional question–answer discussion in class. It also enables the educator to provide immediate feedback to students, which creates an engaging classroom atmosphere and much higher student participation, both on campus and at a distance from campus sites (34).

Hoban et al. used a new form of assessment task, creating blended media, to improve students’ engagement and learning outcomes in pharmacology teaching [55]. Exams, lab reports, and presentations are characteristic assessment tasks for pharmacology courses. In their study, they present an opportunity for students to create a 4–5 min digital media piece analyzing a journal article on a disease related to pharmacology, and further describe the key aspects of that paper. This new form of blended media, student-generated media, permits the students to utilize their own technology to construct a media product which is related to their project, explaining pharmacological scientific concepts [55]. Since presentations require a lot of preparation, this approach possibly, in addition to improving the student’s presentation skills, also permits a deeper understanding of the subject.

The integration of social media within pharmacy schools has been shown to promote marketing, recruitment and student engagement. According to a study by Chen and DiVall (2018), Facebook can be used to post livestream videos and long text posts, as it is a network which a vast majority of students use [56]. However, Twitter was suggested as the best way to communicate and share news with students, and Instagram when the institute wants to share visual content [56].

A separate study revealed that game-based learning provides an opportunity for pharmacy students to experiment with knowledge, increase motivation, and think objectively, and also can provide them with the opportunity to experience things in a “virtual world”. According to the study, the participating pharmacy students preferred role-playing and strategy games on mobiles phones in comparison to racing or sports types of game [18].

## 7. Conclusions

Recently, many universities, faculties, institutions and educators in all disciplines are trying to enhance their quality of teaching, as students demand support and successes in their studies. Pharmacology, which is a vital topic in health care and biosciences programs, can possibly be problematic for students. Therefore, a diversity of teaching methods and strategies are required to inspire student engagement in this challenging course.

Numerous reports have expressed concerns regarding registered nurses’ lack of knowledge in pharmacology and their inadequate knowledge of pharmacotherapy, contributing to medication errors [57,58,59]. It is worth noting that, despite practicing nurses monitoring the drug administration to patients, nursing students are not taught theoretical pharmacological understanding to a great extent, including bioscience [60,61].

Medication errors cause thousands of deaths annually across the globe [41,62,63,64]. The UK government states that one medication error is made for every five drugs handed out, adding up to 237 million errors per year and causing nearly 2000 deaths [63]. In the US, the FDA receives more than 100,000 cases every year of suspected medication error. Therefore, knowledge of medicine and training in prescribing medication are essential [65]. Moreover, correct drug-dose calculation is an essential skill to prevent and reduce medication errors, which is a key element in patient safety [66]. The question that remains to be answered is: do these errors have to do with human errors because of stress and workload or lack of knowledge in terms of medications and pharmacology, or possibly a combination of the two? This essential question needs to be investigated with solid evidence.

To improve pharmacology education, faculties are making a careful consideration of their teaching strategies for every year group, adapting them to the students’ knowledge levels and retention capabilities. Traditional curricula, consisting of lecture-based sessions with a test at the end, are being replaced by curricula involving students choosing what they want to learn to a certain extent, as well as how they will learn and what type of assessments will be given.

Pharmacology teaching entails various topics, such as pharmacodynamics, and pharmacokinetics, with different degrees of difficulty. However, a question which arises, and is necessary to answer is which topics are most suitable for the methods and tools that aim to increase the engagement and activity of the students in pharmacology courses. In general, it is very difficult to conclude and address this question in this work, since research studies were not conducted on a specific topic and the studies did not mention any specific correlation between topics and methods or tools.

In recent decades, amid technological progress in learning tools for teaching, and the cost of distance education courses and students’ requests, the landscape of education has changed and distance learning has skyrocketed. As distance learning provides a unique opportunity, enabling flexibility for both the educator and students, it is necessary to overcome and solve problems such as feeling isolated, and the need for support, technology, time management and discipline.

Collectively, there are different approaches, such as blended learning, FPDT, and applying various instructional strategies such as case studies, humor, and problem-based learning, which can improve student engagement, activity, motivation and learning outcomes in pharmacology courses.

## Figures and Tables

**Table 1:** Improving Student Engagement and Learning Outcomes in Pharmacology Course.

Author	Profession	Major Findings	Ref.
**Sáiz-Manzanares et al.**	Nursing	Blended learning favors greater achievement of effective learning when implemented with Learning Management System (LMS) together with hypermedia resources.	[25]
**Thomas & Schuessler**	Nursing	The student engagement, learning outcomes, and the exam scores were improved by applying various instructional strategies such as games, case studies, and humor.	[26]
**Kaylor**	Nursing	The cognitive load theory (CLT) as an instructional design framework that can be used to overcome the challenges for both nursing students and the educators	[27]
**McEnroe-Petitte**	Nursing	Gaming enhances teaching and remediation of content as well as being fun and promoting student engagement.	[11]
**Oyler et al.**	Pharmacy	A “chunk and chew” strategy, that is, splitting a subject into subtopics (chunks) and permitting the students to process (chew) the material in groups between sections.	[28]
**Betharia**	Pharmacy	In addition to traditional PowerPoint slides, utilizing the active development of concept diagrams will encourage student engagement and enhance the understanding of content in pharmacology lectures.	[29]
**Zgheib et al.**	Medical	Using team-based learning (TBL) to teach pharmacology improved the group performance compared with individual performance, however, if questions were particularly difficult then it was less successful.	[30]
**Kerecsen & Pazdernik**	Medical	The use of computer-based pharmacology systems requires constant updates with respect to both content and changing technologies.	[31]
**Morton et al.**	Medical	Blended learning combines online digital media with traditional classroom methods; this sort of teaching is more beneficial and helpful rather than consisting solely of online learning.	[32]
**Quesnelle & Montemayor**	Medical	The use of Facebook can create virtual learning communities that can combine formal and informal learning.	[33]
**Bakkum et al.**	Pharmacology program	Kahoot is an interactive game in which the instructor can create a number of multiple-choice questions which are relevant to the learning objectives.	[10]
**Sumanasekera et al.**	Pharmacology program	Using Kahoot to learn pharmacological concepts was the most valuable active learning strategy compared to fill-in-the-blank activities and videos.	[15]
**Dos Reis Lívero et al.**	Pharmacology program	Games such as word games, puzzles, tic-tac-toe and others improve pharmacology knowledge.	[13]
**Nakagawa & Yamashita**	Pharmacology program	An additional useful tool when teaching pharmacology is implementing audience response systems (ARS).	[12]

## Data Availability

Not applicable.
